# Supplementary material for: Changes in the three-dimensional microscale topography of human skin with aging impact its mechanical and tribological behavior
Source: PLoS One. 2021 Jul 9;16(7):e0241533. doi: 10.1371/journal.pone.0241533 (PMC8270165; doi:10.1371/journal.pone.0241533)
Supplement: S1 File — (PDF) [file pone.0241533.s001.pdf]

Changes in the three-dimensional microscale topography of human skin with aging impact its mechanical and tribological behavior

Input files

FEBio files are available at the following link:
https://bitbucket.org/abuganzatepole/skinmicrorelief3dmodel

Principal strains during skin indentation

The main text shows the normal strains in a cross section of the domain as skin is indented (Figure 3). In the supplement we further show the first principal Green-Lagrange strain $\boldsymbol{E1}$, as well as the components $\boldsymbol{E_{11}}, \boldsymbol{E_{12}}$ to further illustrate the role of microrelief and individual skin layer properties on the strain distributions over a central cross-section of the three-dimensional domain. Figure S1 shows that, in the wet condition, for which the top layer of the skin is soft compared to the underlying layer, the epidermis, indention of a flat geometry leads to strains which increase gradually over the epidermis and are largest in the dermis, after which they decrease over the hypodermis. Accounting for microrelief realds to higher strains which propagate all the way down to the bottom of the domain. The reason for this increase in strain is that the microrelief geometry consists of peaks and valleys, thus, even though the distance of the indenter to the equivalent flat skin surface remains the same, the microrelief has features above this line which interact with the indenter sooner with respect to the flat case. In the dry condition, characterized by stiffer SC, the strains are higher compared to the wet SC.

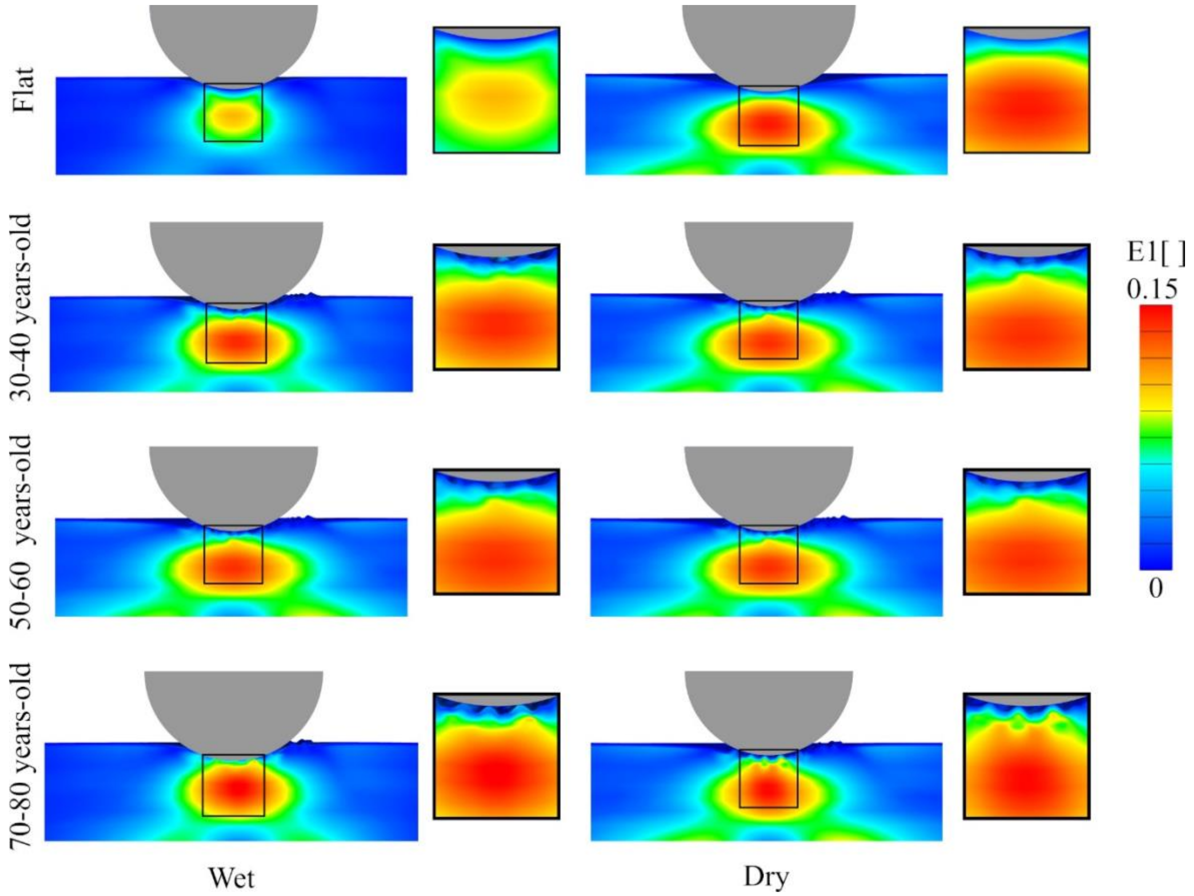

Figure S1 E1 for different SC condition (wet or dry) for the three different microrelief geometries and the flat control.

The contours of the E_{11} component of the Green-Lagrange strain in Figure S2 show much less influence from microrelief changes or changes of SC properties between wet and dry conditions compared to Figure S1 or Figure 3 in the main text. Indeed, the indentation step mostly affects the normal strain component or the first principal strain. Similarly, Figure S3 shows that the shear strain component is minimally affected by the changes in microrelief or changes in SC properties. Nevertheless, Figure S2 does show that the microrelief leads to strain concentration around the microscale features, mostly in the epidermis, which might be important for improving understanding of epidermis mechanosensing.

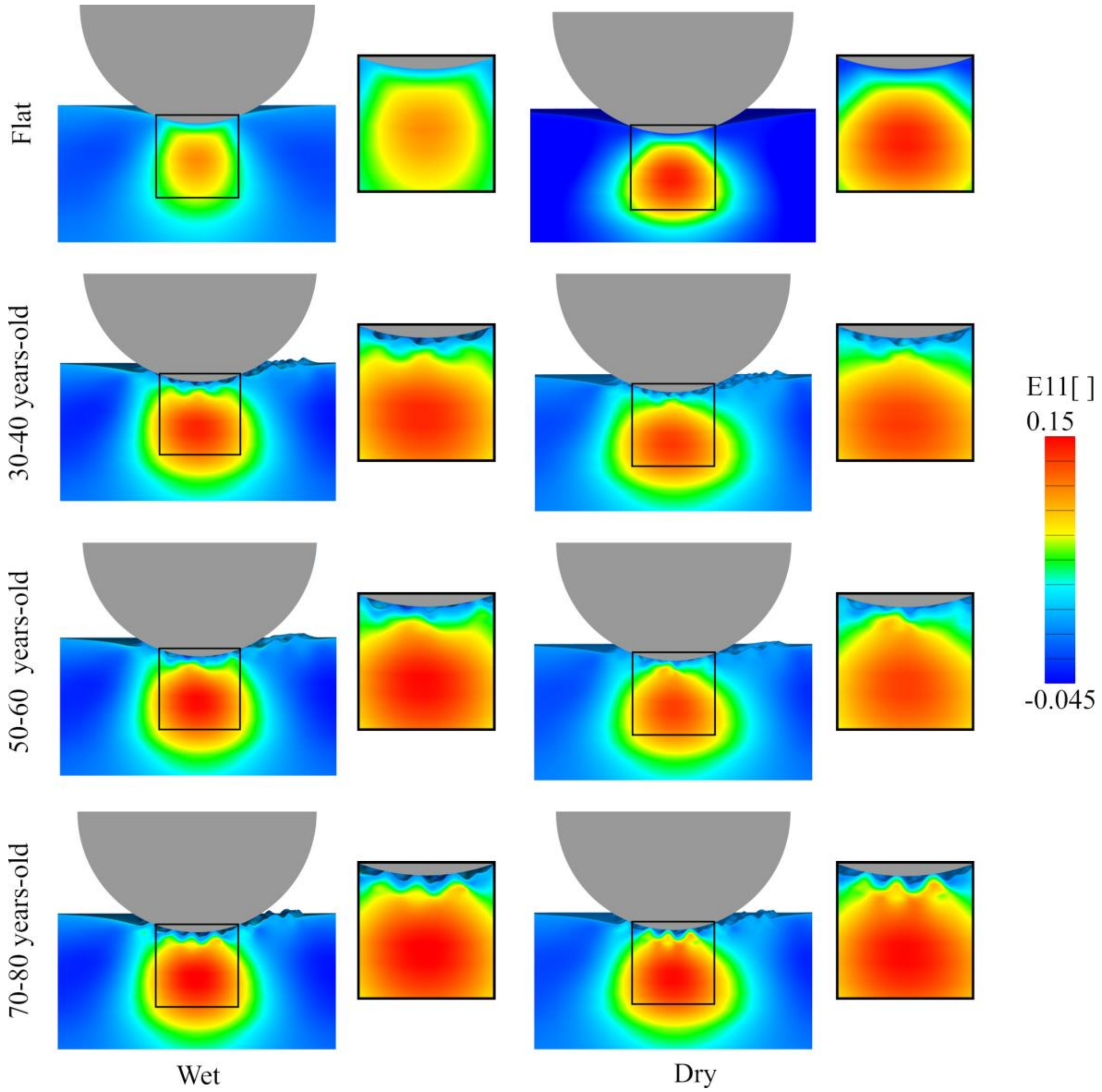

Figure S2 E11 for different SC condition (wet or dry) for the three different microrelief geometries and the flat control.

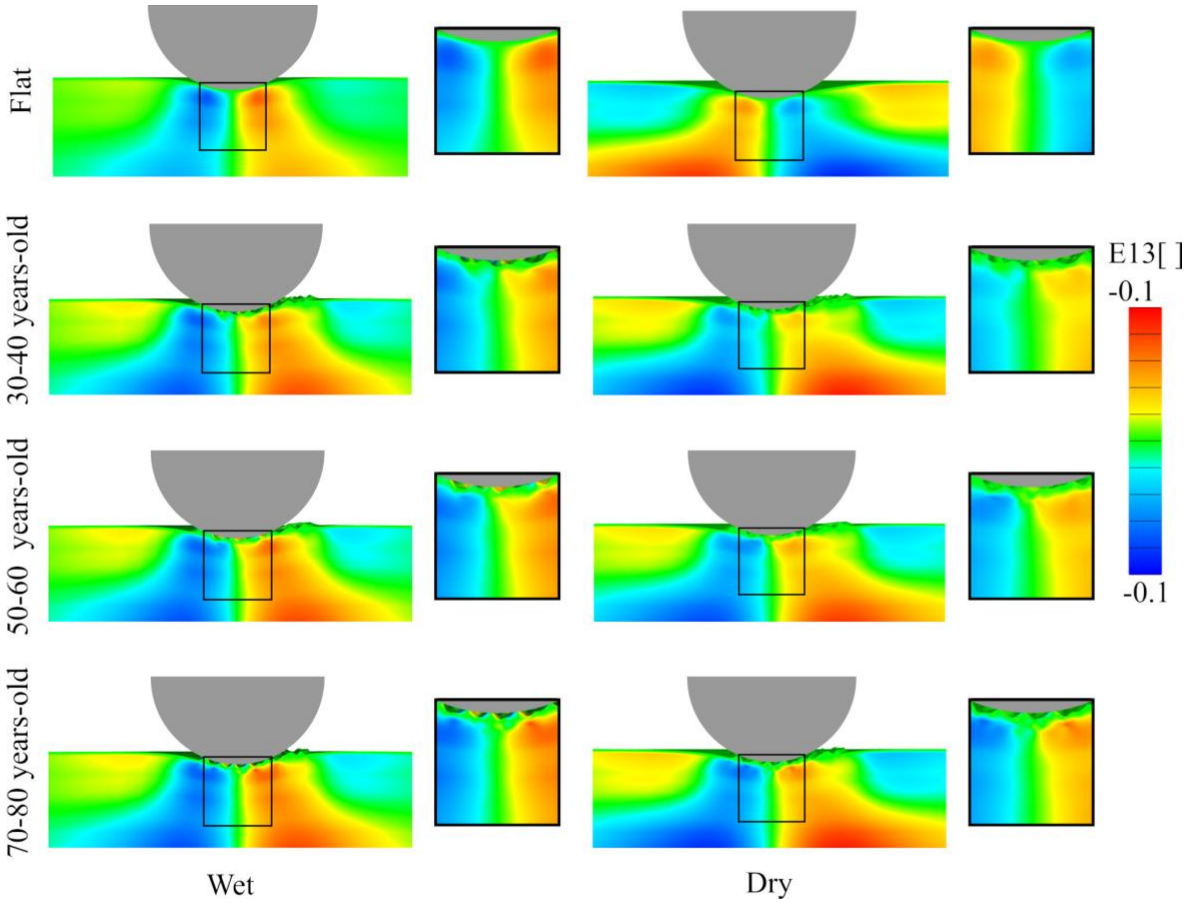

Figure S3 E13 for different SC condition (wet or dry) for the three different microrelief geometries and the flat control.

Green Lagrange Strain components plotted across the thickness

The main text shows the E_{33} and E13 Components of the Green-Lagrange plotted across the skin thickness right underneath the indenter for the different age topographies and different SC condition (wet or dry) (Figure 4). In the supplement we present these components as well as the first principal Green-Lagrange strain E_1, in different positions: right underneath the indenter apex, R/2 and R from indenter apex. Results are for the 50-60 years old topography. A minor variation in the trend is observed between dry (dashes) and wet (solid line) SC conditions. The main noticeable difference among the dermal stiffness is the E13 component in dermis zone.

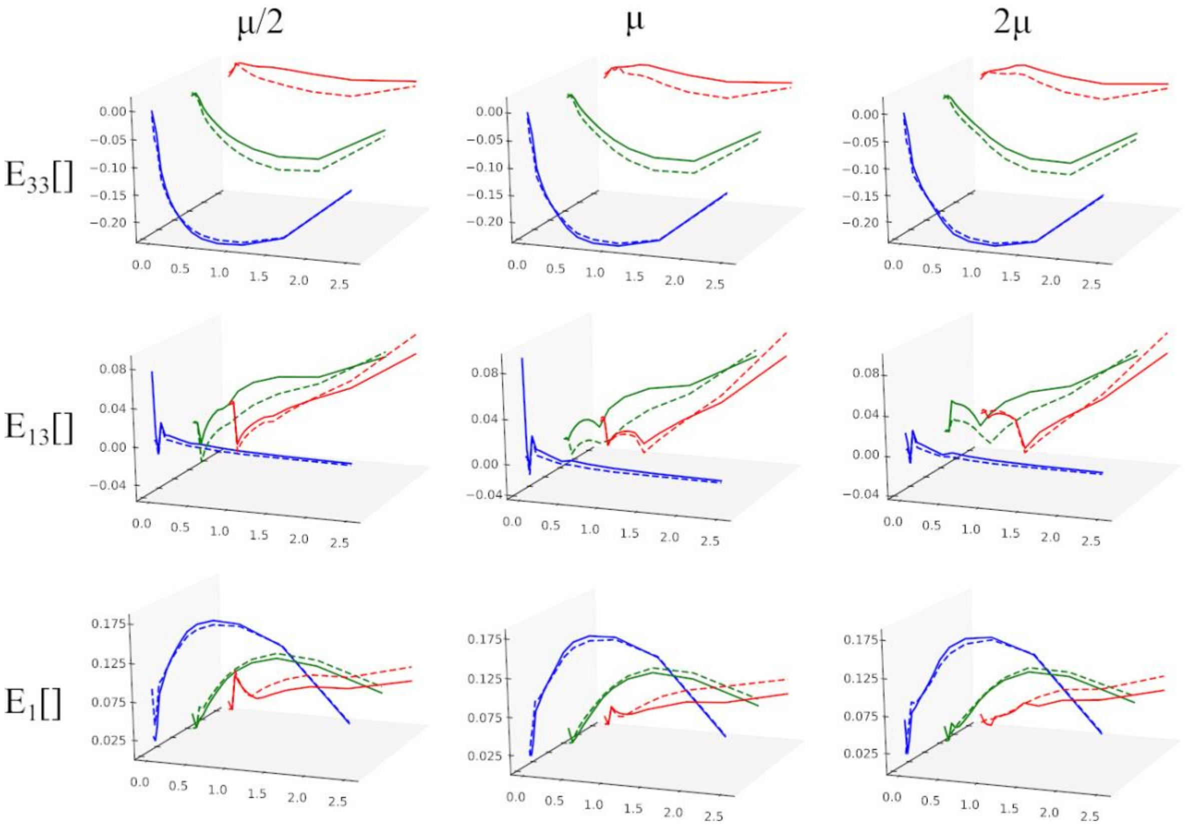

Figure S4 50-60 years-old topography E_{33}, E_{13} and E_1 plotted across the skin thickness for in three different positions underneath the indenter. Blue, green and red correspond to underneath indenter apex, R/2 and R away from the center respectively. The solid line represents the wet SC case and dashes are for the dry SC simulations.

E33 Green Lagrange Strain component and surface stress during indenter movement
The main text shows the maximum principal stress at the skin surface when the indenter moves in the anatomical directions U_1 which corresponds to the lateral axis. Figure S5 shows the maximum principal stress when the indenter moves in the directions given by the primary skin lines U_3 and U_4. Similar to the discussion in the main text, the skin microrelief becomes more anisotropic with aging, which leads to an increasingly anisotropic stress profile at the surface as the skin interacts with the indenter.

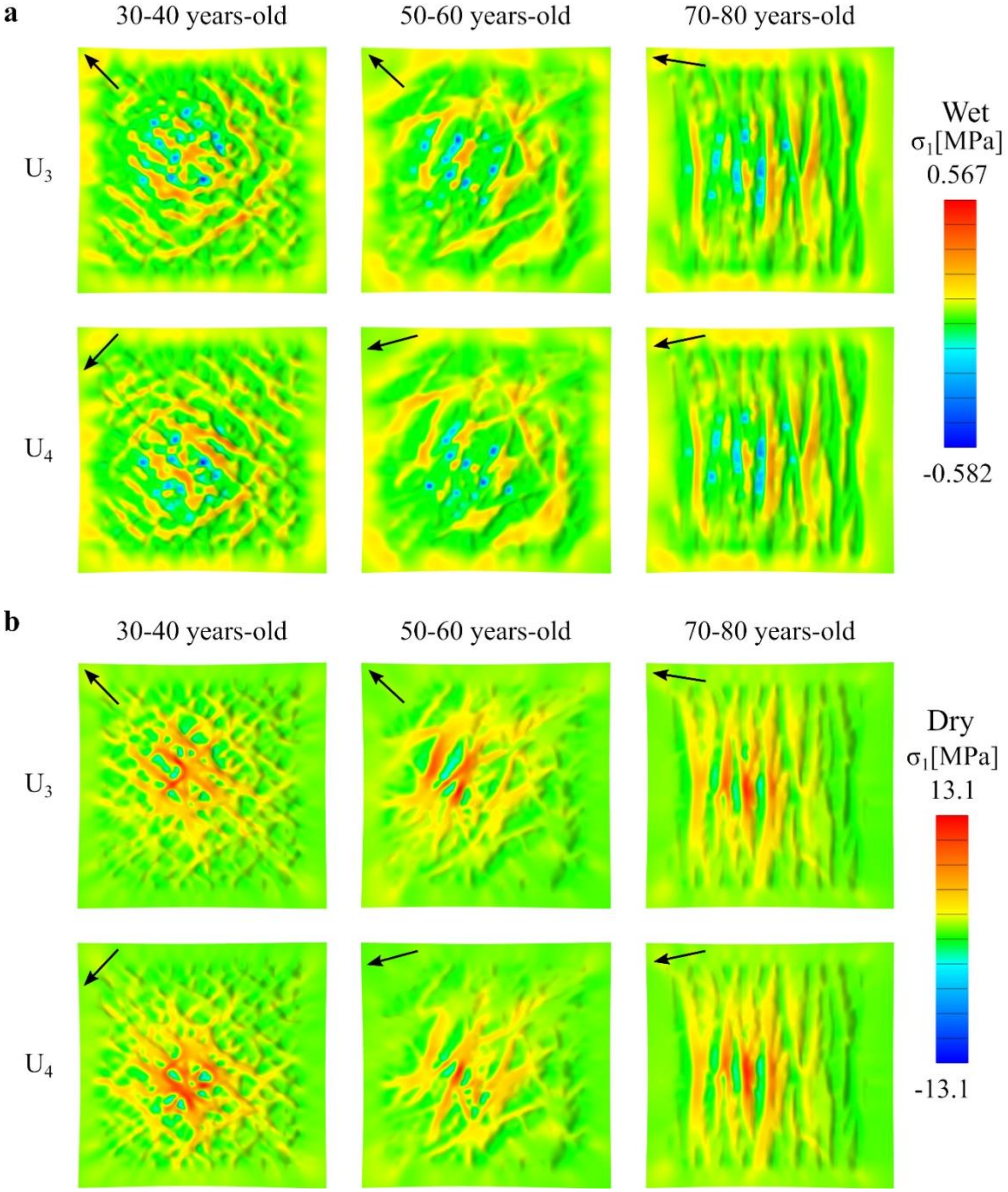

Figure S5 Maximum principal stress contours for three different skin age topographies, for the displacement directions U3 and U4 given by the primary skin lines. The results correspond to: a) wet SC b) dry SC material properties.

E33 Green Lagrange Strain component and surface stress under intermediate SC mechanical properties

The values for SC mechanical properties considered in the main text are at the extremes of the values reported in the literature. Therefore, we expected that our results in the main text would reflect these extreme conditions. To further confirm that an intermediate SC stiffness [E=100 MPa] would show an intermediate response with respect to our main results, we ran additional simulations in the 30-40 years-old topography. Figure S6 show the contours of E33 Green Lagrange Strain component for all SC conditions and figure S7 the maximum shear at the top surface. As expected, this intermediate SC stiffness leads to results in between wet and dry conditions reported in the main text.

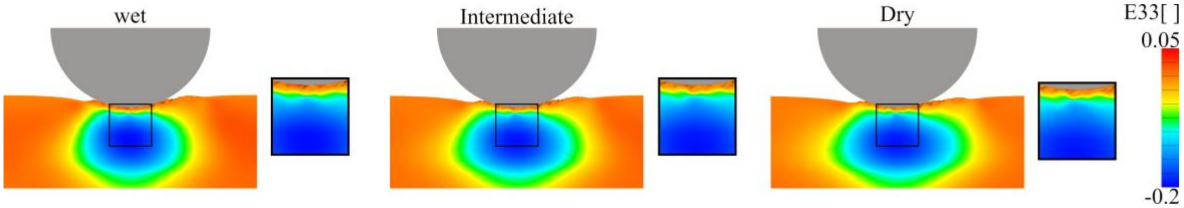

Figure S6 E33 component of the Green Lagrange Strain plotted across the thickness for different SC condition: wet, intermediate and dry for SC with intermediate stiffness of E=100MPa.

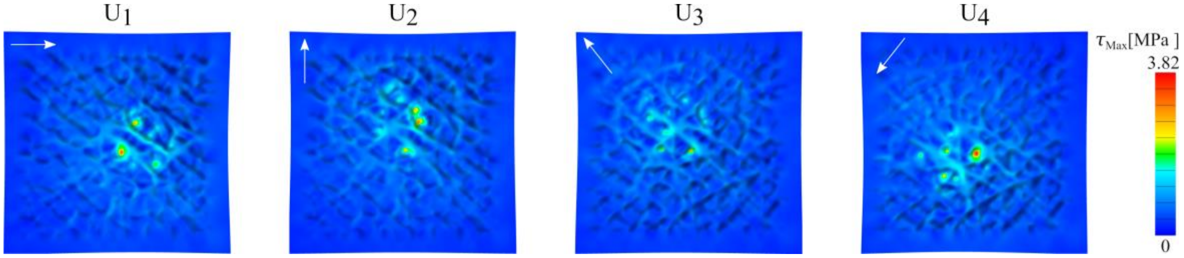

Figure S7 Maximum principal stress contours for intermediate SC condition for all displacement directions. The contours correspond to the final position of the indenter after it has moved in each direction 1 mm. The simulations correspond to an intermediate value of SC stiffness (E=100MPa) with respect to the cases shown in the main text.

Regression for skin friction as a function of model parameters

As indicated in eq. (2), we perform a simple multi-linear regression of the global coefficient of friction in terms of the parameters of the model. The corresponding R functions are results are illustrated here. As can be seen from the results, only the direction of indenter movement with respect to the skin microrelief lines show statistical significance for the global coefficient of friction, whereas neither age nor SC properties led to statistically significant results. A second linear regression was then performed, ignoring all parameters except for direction. These results are shown in Table S2.

Table S1 Generalized linear model for all variables

```
Call:
glm(formula = tableFriction$GCOF ~ tableFriction$Age + tableFriction$SC + tableFriction$Direction, family = gaussian)

Deviance Residuals:
      Min         1Q     Median         3Q        Max
```

```
-0.060141  -0.007372  -0.001449   0.008028   0.069904

Coefficients:
                           Estimate Std. Error t value Pr(>|t|)
(Intercept)               0.2107849  0.0011905 177.050   <2e-16 ***
tableFriction$Age         0.0001986  0.0003715   0.535    0.593
tableFriction$SC          0.0010778  0.0007171   1.503    0.133
tableFriction$Direction  -0.0032078  0.0003206 -10.006   <2e-16 ***
---
Signif. codes:  0 ‘***’ 0.001 ‘**’ 0.01 ‘*’ 0.05 ‘.’ 0.1 ‘ ’ 1

(Dispersion parameter for gaussian family taken to be 0.0003155185)

    Null deviance: 0.81115  on 2468  degrees of freedom
Residual deviance: 0.77775  on 2465  degrees of freedom
AIC: -12891

Number of Fisher Scoring iterations: 2
```

Table S2 Generalized linear model for Direction

```
Call:
glm(formula = table$GCOF ~ table$Direction, family = gaussian)

Deviance Residuals:
       Min          1Q      Median          3Q         Max
-0.0177857  -0.0055959  -0.0008867   0.0058259   0.0202161

Coefficients:
                 Estimate Std. Error t value Pr(>|t|)
(Intercept)      0.214449   0.004695   45.68   <2e-16 ***
table$Direction -0.004046   0.001714   -2.36   0.0275 *
---
Signif. codes:  0 ‘***’ 0.001 ‘**’ 0.01 ‘*’ 0.05 ‘.’ 0.1 ‘ ’ 1

(Dispersion parameter for gaussian family taken to be 8.817396e-05)

    Null deviance: 0.0024310  on 23  degrees of freedom
Residual deviance: 0.0019398  on 22  degrees of freedom
AIC: -152.05

Number of Fisher Scoring iterations: 2
```

Mesh convergence

To verify that the mesh density was accurate we performed a mesh refinement analysis and found that increasing the mesh by a factor of 1.5 resulted in small variations of the stress features. In particular, the maximum shear stress shown in Figure S8 only increases by 0.8% with the 50% increase in mesh elements. The simulations are already computationally demanding with the *coarse* mesh which consists of approximately 350,000 hex elements.

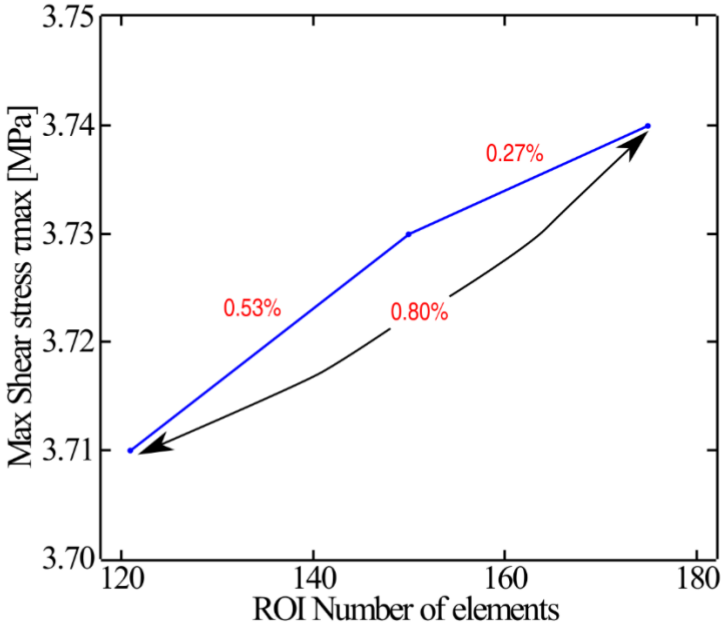

Figure S8 Mesh convergence analysis for Max Shear stress τ_{max} of skin flat topography. The mesh is based on Preview Butterfly 2D 120,150,175 elements in x and y direction for 3.5 mm square ROI.
